# Supplementary material for: Crawling and Gliding: A Computational Model for Shape-Driven Cell Migration
Source: PLoS Comput Biol. 2015 Oct 21;11(10):e1004280. doi: 10.1371/journal.pcbi.1004280 (PMC4619082; doi:10.1371/journal.pcbi.1004280)
Supplement: S1 Code — (ZIP) [file pcbi.1004280.s012.zip › release/tst/doc/html/sticky_8h.html]

Tissue Simulation Toolkit: sticky.h File Reference


Tissue Simulation Toolkit
 0.1.4.1


- Main Page
- Namespaces
- Classes
- Files

- File List
- File Members

Macros |
Functions

sticky.h File Reference

This graph shows which files directly or indirectly include this file:

Go to the source code of this file.

|  |  |
| --- | --- |
| Macros | |
| #define | GRIDX   100 |
|  | |
| #define | GRIDY   100 |
|  | |
| #define | MAXCELLS   1024 /\* beware \*/ |
|  | |
| #define | MAXNEIGH   2054 |
|  | |
| #define | FALSE   0 |
|  | |
| #define | TRUE   1 |
|  | |
| #define | OK   1 |
|  | |
| #define | TESTCELLS   1 |
|  | |
| #define | BOLTZMANN   1024 |
|  | |
| #define | MAXSEED   65531349 |
|  | |
| #define | MAXHIST   4096 |
|  | |
| #define | MAXTYPE   256 |
|  | |
| #define | WHITE   0 |
|  | |
| #define | BLACK   1 |
|  | |
| #define | RED   2 |
|  | |
| #define | BLUE   3 |
|  | |
| #define | GREEN   4 |
|  | |
| #define | MEDIUM   0 |
|  | |
| #define | EMPTY   -1 |
|  | |
| #define | DIV   1 /\* DIV 2^(# of divbit) \*/ |
|  | |
| #define | REMARK   56 |
|  | |
| #define | HASHCOLPRIME   255 |
|  | |
| #define | PLOTPERIODFREQUENCY   25 |
|  | |
| #define | HOSTDEAD   -20 |
|  | |
| #define | NULL\_BEAST   -10 |
|  | |
| #define | OK\_BEAST   -5 |
|  | |
| #define | PMUT   0.009 |
|  | |
| #define | PMUT2   .1 |
|  | |
| #define | DMUT   0.0021 |
|  | |
| #define | PCO   0. |
|  | |
| #define | NETSIZE   29 |
|  | |
| #define | INETSIZE   16 |
|  | |
| #define | INP\_PROT   16 /\* number of negative inputs (connectivity) \*/ |
|  | |
| #define | POTPROT   16 /\* number of potential proteins \*/ |
|  | |
| #define | COMMUNICATION   2 |
|  | |
| #define | ENERGYOFFSET   8 |
|  | |
| #define | NHHIST   10 |
|  | |
| #define | NH\_TH   0.5 |
|  | |
| #define | ENDOFSTATES   1<<(NETSIZE+2) |
|  | |

|  |  |
| --- | --- |
| Functions | |
| double | RANDOM () |
|  | |

## Macro Definition Documentation

#define BLACK   1

#define BLUE   3

#define BOLTZMANN   1024

#define COMMUNICATION   2

#define DIV   1 /\* DIV 2^(# of divbit) \*/

#define DMUT   0.0021

#define EMPTY   -1

Referenced by Cell::ClearJ(), and CellularPotts::SearchNandPlot().

#define ENDOFSTATES   1<<(NETSIZE+2)

#define ENERGYOFFSET   8

#define FALSE   0

#define GREEN   4

#define GRIDX   100

#define GRIDY   100

#define HASHCOLPRIME   255

#define HOSTDEAD   -20

#define INETSIZE   16

#define INP\_PROT   16 /\* number of negative inputs (connectivity) \*/

#define MAXCELLS   1024 /\* beware \*/

#define MAXHIST   4096

#define MAXNEIGH   2054

#define MAXSEED   65531349

#define MAXTYPE   256

#define MEDIUM   0

#define NETSIZE   29

#define NH\_TH   0.5

#define NHHIST   10

#define NULL\_BEAST   -10

#define OK   1

#define OK\_BEAST   -5

#define PCO   0.

#define PLOTPERIODFREQUENCY   25

#define PMUT   0.009

#define PMUT2   .1

#define POTPROT   16 /\* number of potential proteins \*/

#define RED   2

#define REMARK   56

#define TESTCELLS   1

#define TRUE   1

#define WHITE   0

## Function Documentation

|  |  |  |  |  |  |
| --- | --- | --- | --- | --- | --- |
| double RANDOM | ( | void |  | ) |  |

Returns
:   A random double between 0 and 1

References FAC, MBIG, MSEED, and MZ.

Referenced by CellularPotts::AmoebaeMove(), CellularPotts::GrowInCells(), RandomNumber(), and Seed().


---

Generated on Thu Aug 14 2014 22:04:01 for Tissue Simulation Toolkit by  

 1.8.6
